# Burden of Oral Diseases and Access to Oral Care in an Ageing Society

**DOI:** 10.1016/j.identj.2022.06.012

**Published:** 2022-08-25

**Authors:** Jun Aida, Kenji Takeuchi, Michiko Furuta, Kanade Ito, Yuji Kabasawa, Georgios Tsakos

**Affiliations:** a Department of Oral Health Promotion, Graduate School of Medical and Dental Sciences, Tokyo Medical and Dental University, Tokyo, Japan; b Department of International and Community Oral Health, Tohoku University Graduate School of Dentistry, Sendai, Japan; c Division of Regional Community Development, Liaison Center for Innovative Dentistry, Tohoku University Graduate School of Dentistry, Sendai, Japan; d Section of Preventive and Public Health Dentistry, Division of Oral Health, Growth and Development, Kyushu University Faculty of Dental Science, Fukuoka, Japan; e Department of Oral Care for Systemic Health Support, Health Sciences and Biomedical Engineering, Graduate School of Medical and Dental Sciences, Tokyo Medical and Dental University, Tokyo, Japan; f Department of Epidemiology and Public Health, University College London, London, United Kingdom

**Keywords:** Oral care, Older persons, Burden of oral diseases, Social determinants of health, Health economics

## Abstract

**Objective:**

The total years lived with disability among older people, and the concomitant burden of tooth loss in ageing societies have increased. This study is an overview of the burden of oral diseases and access to oral care in an ageing society.

**Methods:**

We selected key issues related to the burden of oral diseases and access to oral care and reviewed the relevant literature.

**Results:**

The rising number of older people with teeth increases their oral health care needs. To improve access to oral care, affordability of care is a great concern with respect to universal health coverage. In addition, accessibility is a crucial issue, particularly for vulnerable older adults. To improve oral care access, attempts to integrate oral health care into general care are being made in ageing countries. For this purpose, provision of professional oral care at home through domiciliary visits and provision of daily oral health care by non-dental professional caregivers are important. Oral health care for older people reduces general diseases such as pneumonia and malnutrition, which in turn could reduce further healthcare costs.

**Conclusions:**

To address the growing burden of oral care in ageing societies, special provision of oral health care to vulnerable older people, and integration of oral care with primary care will be required.

## Burden of oral diseases of older persons

The demographic transition towards ageing societies and the related health burden have triggered a global focus on ageing and health and a fundamental shift in how we think about ageing, bringing forward the concepts of functional ability and participation in society.^1^ In oral health, an epidemiologic transition has further compounded the impact of the demographic transition, with profound implications for the burden of oral conditions on older populations. Oral diseases are one of the most prevalent health conditions globally, and their burden, especially tooth loss, is huge amongst the older population.^2^

Despite the overall improvement of oral health shown by the age-standardised prevalence, the increase in the older population raises the number of people living with oral diseases. When evaluating the burden of oral disease in the older population, it is important to recognise the difference between crude prevalence and age-standardised prevalence. In many countries, the risk of tooth loss has decreased over the past decades due to improved socioeconomic conditions, lifestyle, and medical standards. This is reflected in the improved age-standardised prevalence of tooth loss. On the other hand, the increase in the older population has resulted in an increase in the number of older people with severe tooth loss. For example, the Global Burden of Disease 2017 Study (GBD 2017 study) reported that between 1990 to 2017, the crude prevalence of tooth loss increased by 75.5%, although age-standardised prevalence decreased by 10.4%.^2^ A substantial number of older adults are still experiencing tooth loss, although the age-standardised prevalence of tooth loss has considerably declined in recent decades in most countries.^2^^,^^3^

At the same time, following the increased numbers of older people with some teeth,^4^ other oral conditions such as root caries, periodontal conditions, partial tooth loss, and dry mouth are also increasingly common problems for older people that affect eating and quality of life of older adults.^3^ For example, the GBD 2017 study reported that the prevalence of untreated caries, periodontal disease, and tooth loss amongst older people exceeded 20% to 30%, respectively.^2^ The retention of natural teeth, many of them already heavily restored, into later in adulthood results in a high burden and complex dental treatment needs for current and future cohorts of older adults. The Figure shows the trend of the percentage and number of people with 19 or fewer remaining teeth amongst Japanese persons aged 75 years or older calculated from the Survey of Dental Diseases^5^ and population census. The percentage has decreased linearly, but the number has not linearly declined because of the increasing numbers amongst the older population. In spite of the recent improvement in the number of remaining teeth, there is a substantial number of people with prosthetic treatment needs.

**Figure:**
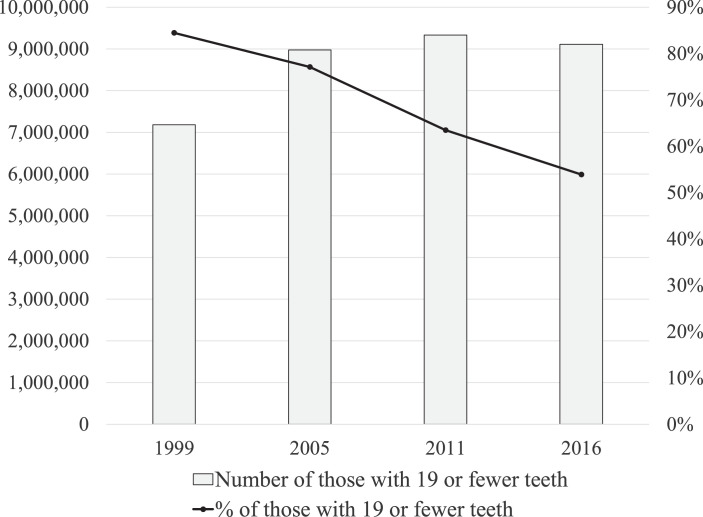
The trend of the percentage and number of people with 19 or fewer remaining teeth amongst Japanese persons aged 75 years or older.

In recent years, a concept has been put forward to summarise poor oral health status amongst older adults: oral frailty. Oral frailty is a frailty phenotype defined as “an age-related gradual loss of oral function together with a decline in cognitive and physical functions.”^6^ With the population ageing, oral frailty has been gaining more attention. A systematic review reported that deterioration of oral health status, especially having a few remaining teeth, was the most associated factor with oral frailty, followed by reduced oral motor skills, including masticatory function and chewing, swallowing, and saliva disorders.^6^ However, there is still a discussion about the definition of oral fraility^7^. There is a concept for categorising the oral health of older people that focuses on the more severe decline of oral function and more diverse oral functions. The Japanese Society of Gerodontology divided oral function into 4 stages from healthier to poorer condition: healthy state, oral frailty, oral hypofunction, and oral dysfunction.^7^ Oral hypofunction is diagnosed and treated by general dentists and meets 3 or more criteria from 7 conditions: poor oral hygiene, oral dryness, reduced occlusal force, decreased tongue-lip motor function, decreased tongue pressure, decreased masticatory function, and deterioration of swallowing function. Oral dysfunction includes eating/swallowing disorder and mastication disorder treated by specialists. To treat these declines in oral function, multidisciplinary cooperation is sometimes required.

Oral diseases were one of the 10 leading causes of total years lived with disability amongst people aged 70 or older in 2019.^8^ Poor oral health status at an older age has negative impacts on our lives in areas such as eating, speaking, smiling, sleeping, and social interactions.^9^ Deterioration of nutrition status and social interactions due to poor oral health can mediate the relation between oral health and general health, such as depression and dementia.^10^^,^^11^ In addition, inadequate oral care can increase the risk of other health problems. Aspiration pneumonia is an important disease associated with oral hygiene amongst older people.^12^ Thus, oral conditions have a considerable impact on the quality of life and well-being of older adults,^13^ frailty^14^ and overall function,^15^ and mortality.^16^ In addition to these impacts of oral health on an individual, oral diseases are also a burden on the population's health due to their high prevalence.^17^ The 74th WHO resolution on oral health described the link between oral health and general health conditions.^18^ Maintaining good oral health is important for older people's nutritional and functional status, and that would reduce mortality and expand healthy life expectancy^19^ amongst older adults.

## Social determinants of oral health throughout the life course

Looking only at the considerable burden of oral conditions on the life of older adults and how to deal with the excessive treatment needs is only one aspect of the whole issue. This burden is not shared equally amongst the different groups in society. Oral conditions are socially patterned, and there is strong evidence of social gradients in oral health amongst older adults.^20^ Importantly, the extent of inequalities in this age group is substantial. For example, adults aged 65 years and older in the poorest quintile in England had almost 8 fewer natural teeth than those in the richest quintile in that age group.^21^ Therefore, it is essential to shift the policy focus towards addressing these excessive and avoidable oral health inequalities.

The theoretical framework of the broader determinants of health^22^ is very helpful in that respect, as oral diseases and inequalities in oral health are caused by a complex array of individual, social, environmental, economic, and political determinants mostly shared with other noncommunicable diseases. Whilst the emphasis has traditionally been on more proximal determinants, such as behavioural factors and dental service use, there is increased understanding that social position (eg, education, income, wealth) can influence health through the material circumstances and psychosocial factors (eg, social capital) with which people live, work, and age. At a broader level, structural determinants of the economic, political, and environmental context (eg, macroeconomic policies, welfare state) play an important role in shaping health (and oral health) inequalities, and these are also considerably affected by the commercial determinants that refer to the “strategies and approaches of the private sector to promote products and choices detrimental to health.”^23^ Understanding these complex interactions amongst the determinants of health can be very beneficial in terms of guiding effective policies to tackle oral health inequalities.

The oral health of older adults reflects their lifelong exposure to oral diseases and oral health care and is affected by the social determinants of health.^24^ Fetal teeth are formed under the influence of factors such as the mother's nutritional status and fluoride levels in the local water supply. After birth, oral health changes due to exposure to various risk and preventive factors such as dietary intake, oral hygiene habits, fluoride exposure, smoking, and access to dental care.^25^ These proximal causes of oral diseases are influenced by upstream social determinants of health throughout the life course. For example, access to dental care at appropriate timing is essential for maintaining oral health and recovering from oral diseases. Individuals’ behaviour for dental care access is affected by the economic status of individuals and the health care system of the society where the individuals live.^26^^,^^27^ Amongst older adults, it is not always easy to receive regular oral care because of their health conditions.^28^ Family or caregivers of disabled older people may not be able to facilitate appropriate oral care or monitor the oral condition. These backgrounds increase the number of older people vulnerable to oral problems.

## Challenges associated with access to oral care for older persons and how to remove barriers to access

Oral care for older adults requires prevention and management of dental caries and periodontal disease, understanding of nutrition and staining dysphagia, and accurate understanding and monitoring of systemic conditions. Access to health care is classically known by the five A's: affordability, availability, accessibility, accommodation, and acceptability.^29^ For older people, any of these factors can be a barrier to access. In countries with ageing populations, the number of disabled older people who have difficulty accessing dental clinics is increasing. In Japan, the number of users of long-term care services increased by 1 million, from 2.78 million in 2009 to 3.78 million in 2019.^30^ In addition, for older people living in care homes, whether the facility provides or makes arrangements for the provision of dental health care services and the attitudes of family members are the major factors in determining access. In Japan, one of the countries with the most advanced ageing population in the world, public insurance, such as universal health care insurance and long-term care insurance, covers oral health care in facilities and home visits by dentists and dental hygienists. This improves access to dental care amongst disabled older adults and helps to reduce the financial burden and secure human resources. However, the situation is quite varied in other countries, and caring for the oral health of vulnerable older adults has only recently been seen as a priority even in affluent countries. In order to promote oral health care in institutions and other settings, it is important to integrate oral health into general primary care, which requires consideration of a variety of factors at the micro to macro level. A review of oral health care for frail older people in Australia recommends multidisciplinary oral health care in institutions and other settings, as well as monitoring of oral health status by nondental professionals. Internationally, there are tools to promote oral health assessment in facilities where dentists are not present. In addition, in Japan, domiciliary dental visits covered by universal health insurance are conducted for older people who need nursing care but are not residents of facilities, which helps address the oral health needs of older people who cannot access dental care in clinics. The following sections provide topics of oral care in 2 ageing countries, and this is followed by a concise overview of the oral health tools available for use amongst care home residents by nondental personnel.

### The situation of oral care amongst older people in Japan

The growing number of older people and number of remaining teeth increase oral health problems amongst older Japanese persons. In Japan, over half (51.2%) of older people have achieved the goal of keeping at least 20 teeth at the age of 80 years. However, there are many oral health problems amongst older adults: caries, mainly caries on the cervical region and root surface, and periodontal disease. In addition, approximately 40% of people aged 70 and older are unable to chew anything.^31^ These results indicate that older people have various oral health care needs, including dental diseases and oral dysfunction. Due to the decline of cognitive function and activities of daily living, toothbrushing habits and visiting the dental clinic are often difficult amongst older people. Moreover, many older people who have other diseases require special consideration for dental treatment due to their health status and medication use.

Japan is a country with a wide coverage of dental care by public insurance,^26^ and this is true even for the treatment of older people. For example, universal health care insurance covers the examination and treatment of oral hypofunction or oral dysfunction as well as home visits for dental treatment and care by a dental hygienist. In addition, public long-term care insurance covers oral health care in facilities. Thus, although delivering dental care in facilities and homes is not easy, the use of these services has increased.

However, although this coverage improves access to dental care, there is also the problem of the availability of dentists who provide more specialised care, such as treatment and rehabilitation of swallowing function in patients with severe oral-stage dysphagia.

### Comprehensive information of oral care for older people to individuals and health care professionals from Australia

Receiving regular professional dental care is difficult for frail older people, especially those living in nursing homes. Therefore, an interdisciplinary approach is required to achieve good oral health. Professionals in Australia summarised the information and useful tools for individuals and health care professions.^32^ In the article, oral health problems and related issues introduced were the relationship between oral and systemic health, dry mouth, dental caries, infection in bone from root stumps after severe dental caries, and oral symptoms due to drug side effects. To tackle these issues, regular assessment of oral health of older people by non–dental health professionals is essential for early referral to a dentist. For this purpose, several assessment methods were introduced. For routine oral care, the article included detailed information on risk-specific oral care methods, a list of appropriate products, as well as videos and information. This comprehensive and detailed information will help nondental professionals and the older people themselves to implement oral health care for older people.

### Oral health tools for older adults in care homes

In addition to dental professionals, nondental professionals are also essential for promoting the oral health of older people. Therefore, it is necessary to use oral assessment tools that can be used by people other than dental professionals in daily settings to correctly identify oral care needs and connect them to dental professionals.^33^ A variety of assessment tools have been developed around the world to help caregivers other than dental professionals quickly recognise changes in the oral health of older people who need care and connect them to dental care. For example, the Oral Health Assessment Tool (OHAT), the Oral Health Screening Tool for Nursing Personnel (OHSTNP), and the Optimized Oral Health-Related Section (ohr-interRAI) are examples of oral assessment tools that have been developed to screen for oral problems in older people who need nursing care.^33^, ^34^, ^35^ In particular, the ohr-interRAI is part of the interRAI, a tool for comprehensively assessing non–oral care needs, and is widely used for holistic care planning in more than 30 countries in North America, Europe, Asia, and the Pacific Rim.^33^ The ohr-interRAI has been developed with the use of photographs and video training for caregivers to improve their ability to detect oral care needs and has been validated; therefore, it is expected to become a more useful oral assessment tool in the future.

## Health economics (cost savings of oral preventive care and financial burden)

### Health care costs savings associated with oral preventive care

Health care costs increase with age, and in developed countries such as the United States^36^ and Japan^37^ they grow faster for the older population than for younger adult populations. Therefore, as health policy focuses on improving the health of the population to reduce the use of limited health care resources,^38^ oral health promotion in older people is considered a way to improve overall health and reduce health care costs.

The variety of oral health problems experienced by older people can result in chewing difficulties that lead to insufficient food intake and subsequently malnutrition.^39^ Malnutrition is associated with declined functional ability, impaired muscle function, immune dysfunction, reduced cognitive function, poor wound healing, higher hospital and readmission rates, and mortality.^40^ Furthermore, oral diseases, especially periodontal disease, are linked with systemic conditions such as diabetes, cardiovascular disease, and pneumonia.^41^ The cumulative evidence confirms that poor oral health contributes to various disease states.

In regard to the association between oral and systemic health, recent studies reported the association between oral health conditions and medical care costs in older people. Older people with a high level of periodontal inflammation had higher inpatient and total medical care costs.^42^ This suggests that an increased risk of inflammation-related disease in individuals with periodontitis results in excess medical care costs.^42^ Moreover, the number of teeth was negatively associated with lower medical care costs related to stroke.^43^ A study of another population showed that a lower number of teeth was associated with more hospitalisation days and higher medical care costs for digestive cancer.^44^ These studies suggest that a lower number of teeth increases the risk of long duration of hospitalisation. Hospitalisation is a large component of medical care costs; therefore, increases in medical care costs in older people with a lower number of teeth appear to be driven primarily by increased hospitalisation risk.

Oral preventive care throughout life is essential to preserve good oral health. Dental care services are known to shift from treatment to preventive care.^45^ These trends are expected to continue, with an increasing demand for preventive care and diminishing demand for treatment.^46^ Even though expenses for oral preventive care are increasing, this increased use of oral preventive care could offset not only dental treatment expenditures but also some medical care costs due to improvement of overall health.^47^ Oral preventive care in older people may be efficacious in reducing health care costs by preventing oral diseases as well as multiple types of systemic deterioration.

### Financial burden

Providing oral health services delivery and access to oral preventive care are essential parts of maintaining oral and systemic health. On average, 60% of adults in the Organisation for Economic Co-operation and Development (OECD) countries in 2014 had at least one dental visit in the past 12 months.^48^ In the United States, the share of the population who visited a dentist was less than half, 41%, compared to 82% in Germany. Germany is one of only 3 OECD countries (Germany, Japan, and the Slovak Republic) where more than half of dental expenditures are covered.^48^ On average, less than one-third of dental expenditures are covered by government schemes or compulsory insurance.^48^ There is an inequality in the utilisation of dental care services, partly due to the financial burden imposed by the costs of dental care caused by the differences in the public coverage for the care.

Socioeconomic inequalities are significant in most OECD countries: There is an almost 20% difference in dental visits between high- and low-income groups.^48^ Just as there are socioeconomic inequalities in oral health,^49^, ^50^, ^51^, ^52^ there are also socioeconomic inequalities in dental visits.^53^, ^54^, ^55^, ^56^ A multicountry study of adults aged 50 years and older from 14 European countries reported significantly higher access to dental care services by individuals in the highest-income group than peers in the lowest-income group.^53^ Older adult population is not immune from this trend.^54^^,^^57^ In addition, a study from Sweden reported that about 60% of the socioeconomic inequalities in poor oral health were explained by lack of access to dental care services.^58^ Thus, socioeconomically disadvantaged people have greater needs for dental care and are less likely to use dental care services, accelerating inequalities in oral health.

In many countries, dental care is provided by both public and private health care systems. Whilst low socioeconomic position and lack of insurance are significant financial barriers to dental care,^59^ differences in the use of public- and private-sector dental care services amongst different socioeconomic groups may also contribute to overall polarisation in the use of dental care. A recent Finnish report^56^ examined how differences in socioeconomic factors are associated with the use of dental care in the public and private sectors. The provision of dental care in Finland is an example of a 2-sector system where dental care provided through the public sector is universally covered for all residents and dental care provided in the private sector is free market–based. There were differences in the overall utilisation of dental care between residents with higher and lower educational attainment and income, indicating that a high socioeconomic position was strongly associated with a higher likelihood of having dental visits.^56^ However, the difference was mainly attributed to visits to private dental clinics, with little association between higher socioeconomic position and use of public dental clinics.^56^ Thus, public coverage of dental services could potentially mitigate, to some extent, the gap in dental visits between high– and low–socioeconomic status groups.

In its most recent statement, the WHO recommended strengthening the provision of oral health services as part of the package of essential health services to achieve universal health coverage.^60^ Expanding the coverage of dental insurance is reported to increase dental service use in the general population, including older adults.^61^^,^^62^ However, it should be noted that the finding is not applicable to all national circumstances. The existence of financial barriers to dental visits, even in a country where dental services are already publicly covered, is an issue that needs to be resolved in order to design an equitable dental health care system.

## Conclusions

Oral health is essential for older people, and oral care needs are growing in ageing societies. Provision of oral health care to vulnerable older people, including care home residents, is required. Integration of oral care with general care will possibly improve the quality of daily life of older people and lower health care costs.

## Conflict of interest

None disclosed.

## References

[1] World Health Organization (2015).

[2] Bernabe E, Marcenes W, G. B. D. Oral Disorders Collaborators (2020). Global, regional, and national levels and trends in burden of oral conditions from 1990 to 2017: a systematic analysis for the global burden of disease 2017 study. J Dent Res.

[3] McKenna G, Tsakos G, Burke F (2020). Managing an ageing population: challenging oral epidemiology. Prim Dent J.

[4] Steele JG, Treasure ET, O'Sullivan I (2012). Adult dental health survey 2009: transformations in British oral health 1968-2009. Br Dent J.

[5] Ministry of Health, Labour and Welfare (2017).

[6] Dibello V, Zupo R, Sardone R (2021). Oral frailty and its determinants in older age: a systematic review. The Lancet Healthy Longevity.

[7] Minakuchi S, Tsuga K, Ikebe K (2018). Oral hypofunction in the older population: position paper of the Japanese Society of Gerodontology in 2016. Gerodontology.

[8] Collaborators GBDA (2022). Global, regional, and national burden of diseases and injuries for adults 70 years and older: systematic analysis for the Global Burden of Disease 2019 study. BMJ.

[9] Tan H, Peres KG, Peres MA. (2016). Retention of teeth and oral health-related quality of life. J Dent Res.

[10] Kusama T, Kiuchi S, Umehara N (2021). The deterioration of oral function and orofacial appearance mediated the relationship between tooth loss and depression among community-dwelling older adults: a JAGES cohort study using causal mediation analysis. J Affect Disord.

[11] Kiuchi S, Cooray U, Kusama T (2021). Oral status and dementia onset: mediation of nutritional and social factors. J Dent Res.

[12] Seitz MW, Listl S, Bartols A (2019). Current knowledge on correlations between highly prevalent dental conditions and chronic diseases: an umbrella review. Prev Chronic Dis.

[13] Rouxel P, Tsakos G, Chandola T (2018). Oral health-a neglected aspect of subjective well-being in later life. J Gerontol B Psychol Sci Soc Sci.

[14] Hakeem FF, Bernabé E, Sabbah W. (2019). Association between oral health and frailty: a systematic review of longitudinal studies. Gerodontology.

[15] Matsuyama Y, Listl S, Jürges H (2021). Causal effect of tooth loss on functional capacity in older adults in England: a natural experiment. J Am Geriatr Soc.

[16] Peng J, Song J, Han J (2019). The relationship between tooth loss and mortality from all causes, cardiovascular diseases, and coronary heart disease in the general population: systematic review and dose-response meta-analysis of prospective cohort studies. Biosci Rep.

[17] Nakazawa N, Kusama T, Cooray U (2022). Large contribution of oral status for death among modifiable risk factors in older adults: the Jages prospective cohort study. J Gerontol A Biol Sci Med Sci.

[18] World Health Organization. World health assembly resolution paves the way for better oral health care. Available from: https://www.who.int/news/item/27-05-2021-world-health-assembly-resolution-paves-the-way-for-better-oral-health-care. Accessed 14 July 2022.

[19] Matsuyama Y, Aida J, Watt RG (2017). Dental status and compression of life expectancy with disability. J Dent Res.

[20] Tsakos G, Demakakos P, Breeze E (2011). Social gradients in oral health in older adults: findings from the English longitudinal survey of aging. Am J Public Health.

[21] Steele J, Shen J, Tsakos G (2015). The interplay between socioeconomic inequalities and clinical oral health. J Dent Res.

[22] Solar O, Irwin A (2007).

[23] Peres MA, Macpherson LMD, Weyant RJ (2019). Oral diseases: a global public health challenge. Lancet.

[24] Celeste RK, Darin-Mattsson A, Lennartsson C (2021). Social mobility and tooth loss: a systematic review and meta-analysis. J Dent Res.

[25] Fisher-Owens SA, Gansky SA, Platt LJ (2007). Influences on children's oral health: a conceptual model. Pediatrics.

[26] Aida J, Fukai K, Watt RG. (2021). Global neglect of dental coverage in universal health coverage systems and Japan's broad coverage. Int Dent J.

[27] Ghanbarzadegan A, Balasubramanian M, Luzzi L (2021). Inequality in dental services: a scoping review on the role of access toward achieving universal health coverage in oral health. BMC Oral Health.

[28] De Visschere LM, van der Putten GJ, Vanobbergen JN (2011). An oral health care guideline for institutionalised older people. Gerodontology.

[29] McLaughlin CG, Wyszewianski L (2002). Access to care: remembering old lessons. Health Serv Res.

[30] Ministry of Health, Labour and Welfare (2020).

[31] Ministry of Health, Labour and Welfare (2020).

[32] Deutsch A, Jay E. (2021). Optimising oral health in frail older people. Australian Prescriber.

[33] Krausch-Hofmann S, Tran TD, Janssens B (2021). Assessment of oral health in older adults by non-dental professional caregivers-development and validation of a photograph-supported oral health-related section for the interRAI suite of instruments. Clin Oral Investig.

[34] Chalmers JM, King PL, Spencer AJ (2005). The oral health assessment tool—validity and reliability. Aust Dent J.

[35] Tsukada S, Ito K, Stegaroiu R (2017). An oral health and function screening tool for nursing personnel of long-term care facilities to identify the need for dentist referral without preliminary training. Gerodontology.

[36] Yang Z, Norton EC, Stearns SC. (2003). Longevity and health care expenditures: the real reasons older people spend more. J Gerontol Series B, Psychol Sci Soc Sci.

[37] Ohmichi H. (1997). [Japanese health care insurance system and its reform]. Nihon Geka Gakkai zasshi.

[38] Fries JF, Koop CE, Beadle CE (1993). Reducing health care costs by reducing the need and demand for medical services. The Health Project Consortium. N Engl J Med.

[39] Azzolino D, Passarelli PC, De Angelis P (2019). Poor oral health as a determinant of malnutrition and sarcopenia. Nutrients.

[40] Chapman IM. (2006). Nutritional disorders in the elderly. Med Clin N Am.

[41] Kuo LC, Polson AM, Kang T. (2008). Associations between periodontal diseases and systemic diseases: a review of the inter-relationships and interactions with diabetes, respiratory diseases, cardiovascular diseases and osteoporosis. Public Health.

[42] Sato M, Iwasaki M, Yoshihara A (2016). Association between periodontitis and medical expenditure in older adults: a 33-month follow-up study. Geriatr Gerontol Int.

[43] Iwasaki M, Sato M, Yoshihara A (2017). Association between tooth loss and medical costs related to stroke in healthy older adults aged over 75 years in Japan. Geriatr Gerontol Int.

[44] Saito M, Shimazaki Y, Nonoyama T (2019). Associations of number of teeth with medical costs and hospitalization duration in an older Japanese population. Geriatr Gerontol Int.

[45] Manski RJ, Macek MD, Brown E (2014). Dental service mix among working-age adults in the United States, 1999 and 2009. J Public Health Dentistry.

[46] Meyerhoefer CD, Panovska I, Manski RJ. (2016).

[47] Huang SS. (2020). Should Medicaid include adult coverage for preventive dental procedures? What evidence is needed?. J Am Dent Assoc.

[48] OECD (2019).

[49] Morita I, Nakagaki H, Yoshii S (2007). Gradients in periodontal status in Japanese employed males. J Clin Periodontol.

[50] Tsakos G, Demakakos P, Breeze E (2011). Social gradients in oral health in older adults: Findings from the English Longitudinal Survey of Aging. Am J Public Health.

[51] Molarius A, Engstrom S, Flink H (2014). Socioeconomic differences in self-rated oral health and dental care utilisation after the dental care reform in 2008 in Sweden. BMC Oral Health.

[52] Schwendicke F, Dörfer CE, Schlattmann P (2015). Socioeconomic inequality and caries: a systematic review and meta-analysis. J Dental Res.

[53] Listl S. (2011). Income-related inequalities in dental service utilization by Europeans aged 50+. J Dent Res.

[54] Lee W, Kim SJ, Albert JM (2014). Community factors predicting dental care utilization among older adults. J Am Dental Assoc.

[55] Kailembo A, Quiñonez C, Lopez Mitnik GV (2018). Income and wealth as correlates of socioeconomic disparity in dentist visits among adults aged 20 years and over in the United States, 2011-2014. BMC Oral Health.

[56] Nurminen M, Blomgren J, Mikkola H. (2021). Socioeconomic differences in utilization of public and private dental care in Finland: register-based evidence on a population aged 25 and over. PloS One.

[57] Li C, Yao NA. (2021). Socio-economic disparities in dental health and dental care utilisation among older Chinese. Int Dental J.

[58] Wamala S, Merlo J, Bostrom G. (2006). Inequity in access to dental care services explains current socioeconomic disparities in oral health: the Swedish National Surveys of Public Health 2004-2005. J Epidemiol Community Health.

[59] Locker D, Maggirias J, Quiñonez C. (2011). Income, dental insurance coverage, and financial barriers to dental care among Canadian adults. J Public Health Dentistry.

[60] World Health Organization. Executive board 148th session, resolutions and decisions annexes. Available from: https://apps.who.int/gb/ebwha/pdf_files/EB148-REC1/B148_REC1-en.pdf#page=17. Accessed 14 July 2022.

[61] Srivastava P, Chen G, Harris A. (2017). Oral health, dental insurance and dental service use in Australia. Health Econ.

[62] Meyerhoefer CD, Zuvekas SH, Farkhad BF (2019). The demand for preventive and restorative dental services among older adults. Health Econ.

